# Nonvisualization of the Internal Carotid Artery on Computed Tomography Angiography: Discussion of Two Cases with Review of Literature

**DOI:** 10.1155/2016/7584384

**Published:** 2016-05-19

**Authors:** Sonal Saran, Rengarajan Rajagopal, Pushpinder S. Khera, Neeraj Mehta

**Affiliations:** Department of Radiology, AIIMS Jodhpur, Jodhpur 342005, India

## Abstract

Nonvisualization of the internal carotid artery (ICA) on cross-sectional imaging studies can be due to congenital (dysgenesis of the ICA) or acquired (complete occlusion of ICA) causes. We report two cases, one with absent carotid canal on bone window setting of computed tomography (CT) suggestive of congenital cause and the other with normal carotid canal, suggesting acquired cause. Development of aortic arches with six pathways of collateral circulation in brain is also discussed.

## 1. Introduction

Nonvisualization of the internal carotid artery (ICA) on cross-sectional imaging studies can be due to dysgenesis of the ICA or due to complete occlusion. In both the cases the clinical possibility ranges from that of an asymptomatic patient to one having transient ischemic attack (TIA) and fatal stroke [1].

**Figure 1:**
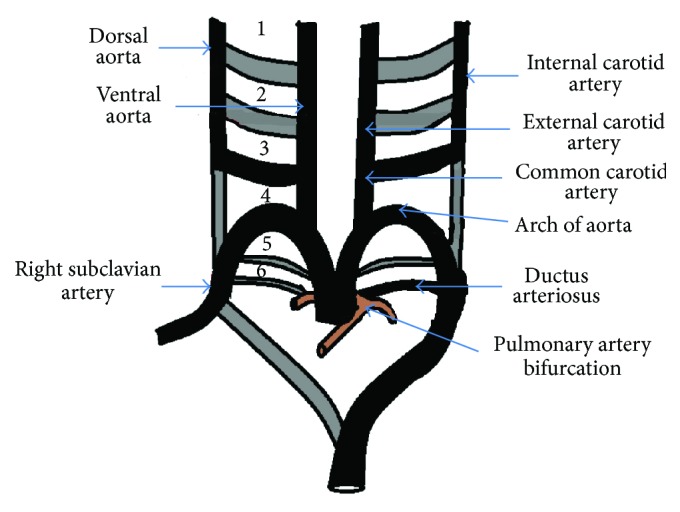
Development of aortic arches is depicted. Numbers one to six represent the aortic arches.

The aorta develops at around 21st day of embryonic life. Primitive aorta consists of ventral and dorsal segments that are continuous through the first aortic arch. The two ventral aortae fuse to form the aortic sac. The dorsal aortae fuse to form the midline descending aorta. Six paired aortic arches (brachial arch arteries) develop between the ventral and dorsal aortae (Figure 1).

*(i) First Arch.* It contributes to the formation of the maxillary and external carotid arteries.

*(ii) Second Arch.* It contributes to the formation of the stapedial arteries.

*(iii) Third Arch (Also Known as Carotid Arch).* Proximal segments of the third pair form the common carotid arteries. The distal portions contribute to the formation of the internal carotid arteries along with segments of the dorsal aortae. The ECA arises as a sprout from the CCA (i.e., the third aortic arch) and also receives contribution from the first and second aortic arches.

*(iv) Fourth Arch.* The left fourth arch forms the aortic arch. The proximal right subclavian artery is formed from the right fourth arch whereas the distal right subclavian artery is derived from a portion of the right dorsal aorta and the right seventh intersegmental artery.

*(v) Fifth Arch.* It forms the rudimentary vessels that regress early.

*(vi) Sixth Arch.* The left sixth arch contributes to the formation of the main and left pulmonary arteries and ductus arteriosus. The right sixth arch contributes to the formation of the right pulmonary artery.

Initially, the aortic arches are connected to the dorsal aorta. As development progresses, the connection of the first and second arches to the dorsal aorta regresses and they contribute to the formation of the ECA. Persistence of the connection with the dorsal aorta may present as transcranial ECA-ICA anastomosis. Through this anastomosis the internal maxillary artery and middle meningeal arteries can supply the distal ICA in cases of hypoplasia of the ICA (known as rete mirabile in the region of the cavernous sinus) [2].

Two longitudinal vascular plexuses dorsal to the third and fourth arches form the basilar artery during the 5th week of intrauterine development. Multiple primitive vessels connect the developing basilar artery and the ICA. All of these vessels involute except for the most cranial one, which persists as the posterior communicating artery [2].

## 2. Case Presentation

We hereby present two cases, which presented to our hospital with symptoms of TIA and, on evaluation with computed tomography angiography (CTA) of carotid vessels, diagnosis of nonvisualization of the ICA was made.

### 2.1. Case One

**Figure 2:**
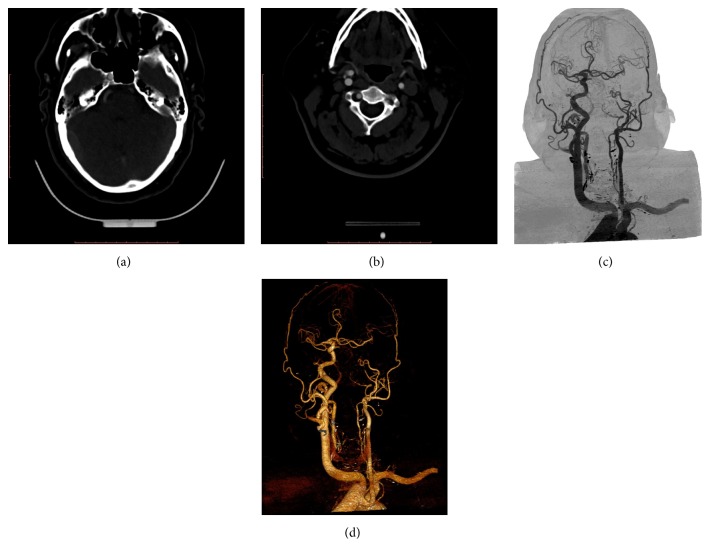
(a) Computed tomographic angiography axial image of the patient shows nonvisualization of the left internal carotid artery with absence of left carotid canal. (b) Computed tomographic angiography axial image of the patient at caudal level shows nonvisualization of the left internal carotid artery. Normal internal and external carotid arteries are seen on the contralateral side. (c) Digital subtraction angiographic image reconstructed by the volumetric rendering techniques shows absent left internal carotid artery with left middle cerebral artery being supplied by the collateral circulation through the anterior cerebral artery and anterior communicating arteries. (d) Three-dimensional reconstruction by the volumetric rendering techniques shows absent left internal carotid artery with left middle cerebral artery being supplied by the collateral circulation through the anterior cerebral artery and anterior communicating arteries.

A 64-year-old male, hypertensive for 20 years (on medication), presented with transient right-sided weakness and numbness. The patient underwent CTA imaging of the carotid vessels and circle of Willis, which showed absent ICA on left side and collateral flow to the left hemisphere through the circle of Willis. Absence of the left carotid canal was also discovered at bone window setting of computed tomography (CT), which confirmed the congenital nature of the nonvisualization of left ICA. Maximum intensity projection (MIP) reconstruction revealed that the left middle cerebral artery was fed through a dilated left anterior cerebral artery supplied by the anterior communicating artery (Figure 2).

There was no associated vascular malformation or any transcranial ECA-ICA anastomosis or any embryonic persistent artery. The patient's symptoms resolved spontaneously and were attributed to either transient ischemic attacks or migraine headaches. No thromboembolic source was identified.

### 2.2. Case Two

**Figure 3:**
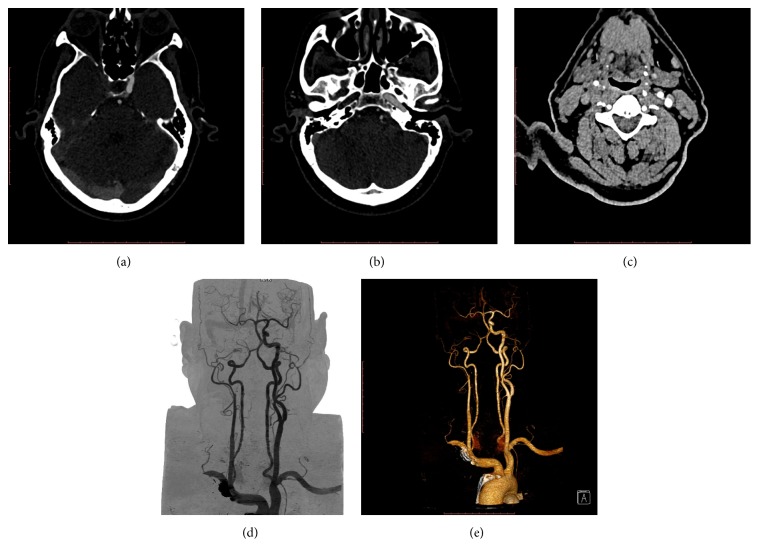
(a) Computed tomographic angiography axial image of the patient shows nonvisualization of the right internal carotid artery. (b) Computed tomographic angiography axial image of the patient shows nonvisualization of the right internal carotid artery with normal right carotid canal. (c) Computed tomographic angiography axial image of the patient at caudal level shows nonvisualization of the right internal carotid artery. Normal internal and external carotid arteries are seen on contralateral side. (d) Digital subtraction angiographic image reconstructed by the volumetric rendering techniques shows absent right internal carotid artery with right middle cerebral artery being supplied by the collateral circulation through the anterior cerebral artery and anterior communicating arteries. (e) Three-dimensional reconstruction by the volumetric rendering techniques shows absent right internal carotid artery with right middle cerebral artery being supplied by the collateral circulation through the anterior cerebral artery and anterior communicating arteries.

A 59-year-old male, hypertensive for 5 years (on medication), presented with transient left-sided weakness and numbness. The patient underwent CTA imaging of the carotid vessels and circle of Willis, which showed nonvisualization of ICA on right side and collateral flow to the right hemisphere through the circle of Willis. Right carotid canal was normal at CTA, which confirmed the acquired nature of the nonvisualization of right ICA. A diagnosis of complete occlusion of the right ICA along its whole course was made (Figure 3).

## 3. Discussion

Our first case was diagnosed as agenesis of the left ICA with absent left carotid canal assessed on bone window setting of CT. Dysgenesis of the ICA includes agenesis, aplasia, and hypoplasia. Complete failure of development of the ICA leads to agenesis whereas hypoplasia refers to a very small caliber ICA after the development started and the term aplasia is used when only vestiges of the ICA are present [3]. Dysgenesis of ICA is a rare congenital anomaly, occurring in less than 0.01% of the population. The left ICA is reported to be affected three times more than the right one as in our case. Most of the patients with dysgenesis of the ICA are asymptomatic. In this setting, the most common type of collateral flow is through the circle of Willis. Secondly, collateral flow can be provided via persistent embryonic vessels or from transcranial collaterals arising from the external carotid artery system [4].

Agenesis of the ICA occurs before 24 mm stage of the embryonic growth [5].

Lie reported the first case of agenesis of the ICA and defined agenesis as the total absence of the entire length of the artery. According to Lie, there are six pathways (types A to F) of collateral circulation associated with agenesis of the ICA. In type A, there is unilateral absence of the ICA with collateral circulation to the ipsilateral anterior cerebral artery and middle cerebral artery through anterior communicating artery and hypertrophic posterior communicating artery, respectively. Unilateral absence of ICA with collateral flow to the ipsilateral anterior cerebral artery and middle cerebral artery across a patent anterior communicating artery comes under type B as in our cases. In type C, bilateral ICA agenesis is associated with patent anastomoses between carotid and vertebra-basilar system. Unilateral agenesis of the cervical portions of the ICA with collateral from an intercavernous communication from the cavernous segment of contralateral ICA comes under type D. In types E and F, there is bilateral ICA hypoplasia with bilateral posterior communicating arteries supplying the middle cerebral arteries in type E and the hypoplastic ICA getting flow from bilateral rete mirabile in type F. Retia mirabilia are transcranial anastomoses between the branches of ICA and external carotid artery system [2].

Congenital absence of ICA is often associated with intracerebral aneurysm formation. The carotid canals in petrous bone form secondary to the presence of the embryonic ICA. Absence or hypoplasia of embryonic ICA leads to hypoplasia of the carotid canal. Absence of carotid canal on a computed tomography scan should suggest a congenital ICA abnormality and suggest an extensive search for associated intracranial vascular malformations [6].

In patients with agenesis of the ICA, cross-sectional imaging techniques are currently the modality of choice [7]. Our second case was diagnosed as complete occlusion of right ICA along its whole course with absolutely normal right carotid canal.

Dysgenesis of the ICA should be differentiated from complete occlusion especially when unilateral. Complete occlusion of the ICA is more likely due to severe atherosclerosis, chronic dissection, or fibromuscular dysplasias [8].

In patients with occlusion of the ICA, postocclusive diminished arterial pressure causes collaterals to develop via the circle of Willis which is important to prevent stroke. The anterior communicating artery and the posterior communicating artery are the collateral channels through which the circle of Willis can supply blood flow to the affected side of the brain. When collateral compensation mechanisms fall short, low-flow infarcts in border zone areas of the brain may develop [9].

Cote et al. evaluated forty-seven patients with ICA occlusion who were asymptomatic or had only mild neurological deficit and prospectively followed them up for an average of 34.4 months. During that period of time, they found that 51% of patients experienced TIAs in the territory of the occluded artery and 23.5% of patients suffered a cerebral infarction [10].

## 4. Conclusion

Agenesis of ICA is mostly asymptomatic, being identified only incidentally. The finding of absent carotid canal on routine CT should suggest the diagnosis. It is important in the management of cerebrovascular accidents as the single ICA supplies both the cerebral hemispheres. ICA dysgenesis has to be distinguished from acquired stenosis as the management of the two conditions is different.
